# From detection accuracy to safety assurance in intelligent plant health early warning systems

**DOI:** 10.3389/fpls.2026.1899310

**Published:** 2026-08-25

**Authors:** Junjie Wei, Haoyang Ren, Jian Miao

**Affiliations:** School of Intelligent Engineering, Shandong Management University, Jinan, China

**Keywords:** decision-support systems, human-in-the-loop decision-making, intelligent phytoprotection, plant disease detection, plant disease forecasting, plant health early warning, plant stress monitoring, response closure

## Abstract

Plant disease and plant stress early warning systems have advanced through deep learning, remote sensing, digital phenotyping, disease forecasting, and sensor networks. Detection accuracy, precision, recall, F1-score, and area under the curve remain indispensable, but they are insufficient for judging whether a warning can support timely and proportionate phytoprotection under field variability. This Mini Review argues that intelligent plant health warning systems should be evaluated not only as prediction models, but also as safety-relevant decision-support systems embedded in biological, agronomic, and operational contexts. We first relate AI-based detection to established plant disease forecasting and decision-support traditions, including weather-based models, epidemiological forecasting, and integrated disease management. We then adapt selected safety-assurance concepts, including risk assessment, failure mode and effects analysis, Bow-tie reasoning, warning-threshold governance, reliability analysis, resilience thinking, and response closure, to host-pathogen-environment warning chains. The proposed framework links AI or sensor outputs with pathogen biology, host susceptibility, environmental conduciveness, inoculum pressure, uncertainty assessment, risk classification, threshold decisions, human or automated verification, intervention, and feedback learning. Illustrative crop-pathogen scenarios, including wheat rust, rice blast, potato late blight, grapevine downy mildew, and citrus greening, show how safety assurance can complement existing forecasting and decision-support systems rather than replace them. The framework remains conceptual, and whether these added assurance functions improve existing warning systems requires comparative evaluation under field conditions. Future systems should be evaluated through detection performance and response-oriented indicators such as lead time, calibration, false-alert burden, missed-warning rate, response completion, disease suppression, economic value, and learning after field action.

## 1 Introduction

Plant disease and plant stress remain persistent threats to crop productivity, food security, and sustainable agricultural systems. Global assessments show that pests and pathogens cause substantial yield losses across major food crops, while climate variability can alter host-pathogen interactions, inoculum pressure, and surveillance difficulty (Strange and Scott, 2005; Garrett et al., 2011; Savary et al., 2019). During the past decade, plant science has increasingly adopted artificial intelligence, UAV and satellite imagery, hyperspectral and multispectral sensing, Internet of Things sensors, and digital phenotyping to detect disease symptoms and stress signals at earlier stages (Furbank and Tester, 2011; Araus and Cairns, 2014; Mahlein, 2016; Sishodia et al., 2020). Recent reviews further show rapid progress in deep learning, remote sensing, and intelligent phytoprotection (Jafar et al., 2024; Shoaib et al., 2025; Upadhyay et al., 2025), while also emphasizing field transferability, data quality, and deployment limitations (Yan et al., 2026; Zhao et al., 2025).

The plant disease warning literature already includes more than image classification. Weather-based models, epidemiological forecasting, disease decision-support systems (DSSs), and integrated disease management have long helped growers decide when to scout, spray, quarantine, or monitor. Classical and modern systems such as BLITECAST, EPIPRE, TOMCAST, BlightPro, vite.net, and rice blast forecasting models show that plant disease warning is an operational decision problem rather than a purely algorithmic classification task (Yuen, 2002; Shtienberg, 2013; Rossi et al., 2014; Small et al., 2015; Nettleton et al., 2019; Helps et al., 2024). Current plant health studies are not limited to accuracy-based evaluation. Nevertheless, many AI-based systems still provide limited guidance on how uncertainty should affect decision thresholds, how alerts should be verified and followed through in practice, and how false or missed warnings should inform subsequent decisions.

Safety-assurance concepts are intended to complement, rather than replace, established approaches in plant pathology, epidemiology, agronomy, machine learning, integrated disease management, and disease decision support. Their contribution lies in making uncertainty, decision thresholds, verification, and follow-up more explicit across the warning process. The review is organized around the chain from biological risk and sensor evidence to decision threshold, verification, intervention, and feedback learning. This framing is intended to address both biological specificity and operational practicality: a warning is useful only when it is grounded in disease biology, feasible for users, and connected to a response that can be checked afterward.

## 2 From detection output to disease-warning decision support

Model performance and warning performance are related but not identical. Model performance asks whether an algorithm assigns correct labels to test samples. Warning performance asks whether a system supports timely, proportionate, and traceable plant health action in a real production environment. A classifier trained on clean images may perform poorly when symptoms are mild, leaves are occluded, pathogens overlap, cultivars differ, or images are collected by non-experts (Barbedo, 2016; Mohanty et al., 2016; Ramcharan et al., 2017). Accuracy-centered evaluation also compresses distinct operational errors: a missed early infection, a false alert, a late alert, and an uncalibrated confidence score have different agronomic consequences. Calibration and uncertainty can further deteriorate under dataset shift (Guo et al., 2017; Ovadia et al., 2019; Gawlikowski et al., 2023).

Established disease forecasting systems provide important lessons for intelligent phytoprotection. BLITECAST and related late blight models used weather and infection-period logic to support fungicide timing; EPIPRE and cereal disease DSSs linked disease observations, crop development, and treatment decisions; TOMCAST and later variants used disease severity values to guide spray intervals; BlightPro integrated weather, host resistance, crop information, and management scenarios for potato and tomato late blight (Yuen, 2002; Shtienberg, 2013; Small et al., 2015). More recent work also compares process-based and machine-learning approaches for rice blast and proposes frameworks for estimating the value of pest-management DSSs when field validation is limited (Nettleton et al., 2019; Helps et al., 2024). AI-based detection is therefore better considered alongside sensing, epidemiological models, and established management protocols rather than as a replacement for disease forecasting.

Established DSSs therefore provide practical comparators for the additional assurance functions proposed here. Existing DSSs already operationalize weather inputs, infection periods, host resistance, disease observations, spray timing, and user-facing recommendations. The added assurance functions focus on failure-mode logging, communication of uncertainty, risk-sensitive threshold review, tiered verification, and tracking whether recommended actions were carried out and what outcomes followed. Established systems such as BlightPro and vite.net therefore provide operational comparators for forecasting, threshold-based recommendations, and field implementation. However, the published systems discussed above do not evaluate the full assurance cycle, including uncertainty handling, verification, response completion, and feedback learning.

A safety-assurance lens adds four questions: what biological and operational uncertainties affect the warning; which threshold turns a detection or forecast into monitoring, verification, or intervention; who or what verifies the signal when expert availability is limited; and whether the response was completed and used for learning. Many DSS studies already include thresholds, economic reasoning, or field validation. The additional contribution proposed here is to organize these components as an assurance cycle that explicitly records failure modes, uncertainty, verification, response, and learning. This matters when AI outputs are added to advisory systems: improved detection may not improve crop protection if it is misaligned with disease development, spray timing, quarantine decisions, or grower capacity to act.

This distinction also helps explain why a safety-assured warning should be evaluated at several levels. A model may be technically robust but operationally weak if it produces too many alerts during periods when growers cannot verify them. Conversely, a model with moderate accuracy may still be useful when embedded in a conservative threshold rule for a fast-moving epidemic with a short treatment window. A further implication is that deployment evidence should not be limited to whether the algorithm detected symptoms. It should also document whether the alert arrived before the biologically relevant decision point, whether the recommended action matched local disease pressure, and whether the user had the resources to confirm and implement that action.

## 3 Adapting safety assurance to biological warning chains

Safety-science concepts cannot be transferred mechanically from industrial systems to plant disease systems. Biological warning chains are shaped by pathogen life cycles, host susceptibility, crop growth stage, inoculum pressure, environmental conduciveness, management history, and climate variability. This biological framing is consistent with disease-triangle reasoning, while extending the warning analysis to crop stage, inoculum pressure, management history, sensing uncertainty, and operational response. Uncertainty may therefore arise not only from image quality, domain shift, or sensor drift, but also from latent infection, mixed stresses, resistant cultivars with weak symptoms, changing pathogen races, delayed ground truth, and weather forecasts that modify infection risk. Climate-driven changes in temperature, humidity, canopy wetness, illumination, and extreme weather can also affect sensor performance, data completeness, and calibration, requiring sensing assumptions and alert thresholds to be reviewed across seasons and operating conditions.

Risk assessment can be adapted by classifying alerts according to likelihood, severity, exposure, and time sensitivity (ISO, 2018; IEC, 2019), but these dimensions must be interpreted through plant-pathological context. A moderate detection probability may justify rapid verification when the pathogen is aggressive, the crop is at a susceptible stage, weather is conducive, and the remaining action window is short. Conversely, weak signals under low environmental risk may require continued monitoring rather than immediate intervention. This interpretation also needs local epidemiological judgement, because the same probability score may have different meanings in seedling production, broad-acre cereal fields, orchards, and protected cultivation.

Failure mode and effects analysis (FMEA) is useful when it maps the warning chain rather than the algorithm alone (IEC, 2018). Failure modes may include non-representative training data, early lesions confused with nutrient deficiency, missing weather or scouting data, sensor drift, weak connectivity, delayed laboratory confirmation, absence of extension support, or field treatment not implemented after an alert. Bow-tie reasoning then links threats, preventive barriers, a missed or late warning, mitigative barriers, and consequences (de Ruijter and Guldenmund, 2016). Threshold governance defines when output should become monitoring, verification, or action; reliability analysis tests dependable operation under specified field conditions; resilience thinking adds fallback monitoring when data or expertise is degraded (Hollnagel et al., 2006; Hollnagel, 2018); and response closure records confirmation, treatment, follow-up inspection, and post-season learning.

These concepts should be adapted proportionally. A smallholder advisory tool cannot require the same audit trail as a regional surveillance platform, and a high-value greenhouse system can justify more intensive sensor redundancy and response documentation than broad-acre monitoring. Safety assurance should therefore be viewed as a scalable set of design questions rather than a fixed engineering template. In low-resource settings, it may start with a simple rule requiring field confirmation for high-risk alerts and end-of-season review of false warnings. In data-rich systems, it may include calibrated uncertainty estimates, automated logs, version-controlled threshold changes, and systematic evaluation of whether warnings changed management outcomes.

## 4 Illustrative crop-pathogen scenarios

The examples below are drawn from published crop-disease and decision-support literature and are intended to illustrate how the framework could be applied in different pathosystems. They do not represent validated implementations. Wheat rust is used as one example, with established DSSs providing practical points of comparison.

In wheat stripe rust early warning, disease can develop rapidly under conducive weather and regional inoculum pressure. A conventional AI workflow might classify UAV, smartphone, or ground images and issue an alert when disease probability exceeds a fixed threshold. A safety-assured workflow treats that output as one part of a risk signal that also includes cultivar susceptibility, crop stage, humidity or rainfall, nearby confirmed cases, and the fungicide or phytosanitary action window (Chen, 2005; Wellings, 2011). Lower verification thresholds may be justified when weather and host susceptibility indicate high false-negative cost. Backup scouting and expert escalation become important when image confidence is low or symptoms resemble nutrient stress.

Rice blast illustrates the need to combine model type, telemetry quality, and biological interpretation. Process-based models and machine-learning models have both been examined for supporting blast management (Nettleton et al., 2019). A safety-assured design would not select a model only by average predictive accuracy. It would also ask whether infection-conducive microclimate, cultivar resistance, leaf wetness or humidity, growth stage, and local validation conditions are represented well enough to support action. In this setting, response closure should record not only whether a warning was confirmed, but also whether fungicide timing, cultivar choice, or water-management advice was feasible for growers.

Potato late blight shows a complementary lesson from established DSSs such as BLITECAST and BlightPro: weather, host resistance, and management tactics can be integrated into location-specific recommendations, but safety assurance would further require explicit tracking of threshold performance, false-negative tolerance, response completion, and economic value (Small et al., 2015; Helps et al., 2024). Grapevine downy mildew and powdery mildew systems show that phenology, canopy humidity, rainfall, cultivar susceptibility, and user adoption shape whether forecasts become timely field decisions (Rossi et al., 2014; Helps et al., 2024). Citrus greening and other vector-associated diseases add another boundary condition: the warning chain may need insect-vector monitoring, regional surveillance, diagnostic confirmation, tree-removal decisions, and grower coordination rather than symptom recognition alone (Gottwald, 2010).

Bacterial wilt and other soilborne or vascular diseases illustrate another boundary condition. Visual symptoms may appear only after substantial internal colonization, and latent infection may occur in planting material; safety assurance may therefore need to emphasize planting-material health, field history, sanitation, soil or water testing, and diagnostic confirmation rather than canopy imaging alone (Dickstein et al., 2024). Across these examples, the transferable element is not a universal threshold rule, but the discipline of making assumptions visible: what evidence produced the signal, which biological factors changed its meaning, what uncertainty remains, what response is proportionate, and what outcome is recorded. This framing allows systems with different crops, pathogens, data richness, and user capacity to be compared through shared assurance questions while preserving disease-specific biology.

## 5 Safety-assured framework, implementation requirements, and metrics

**Figure 1:**
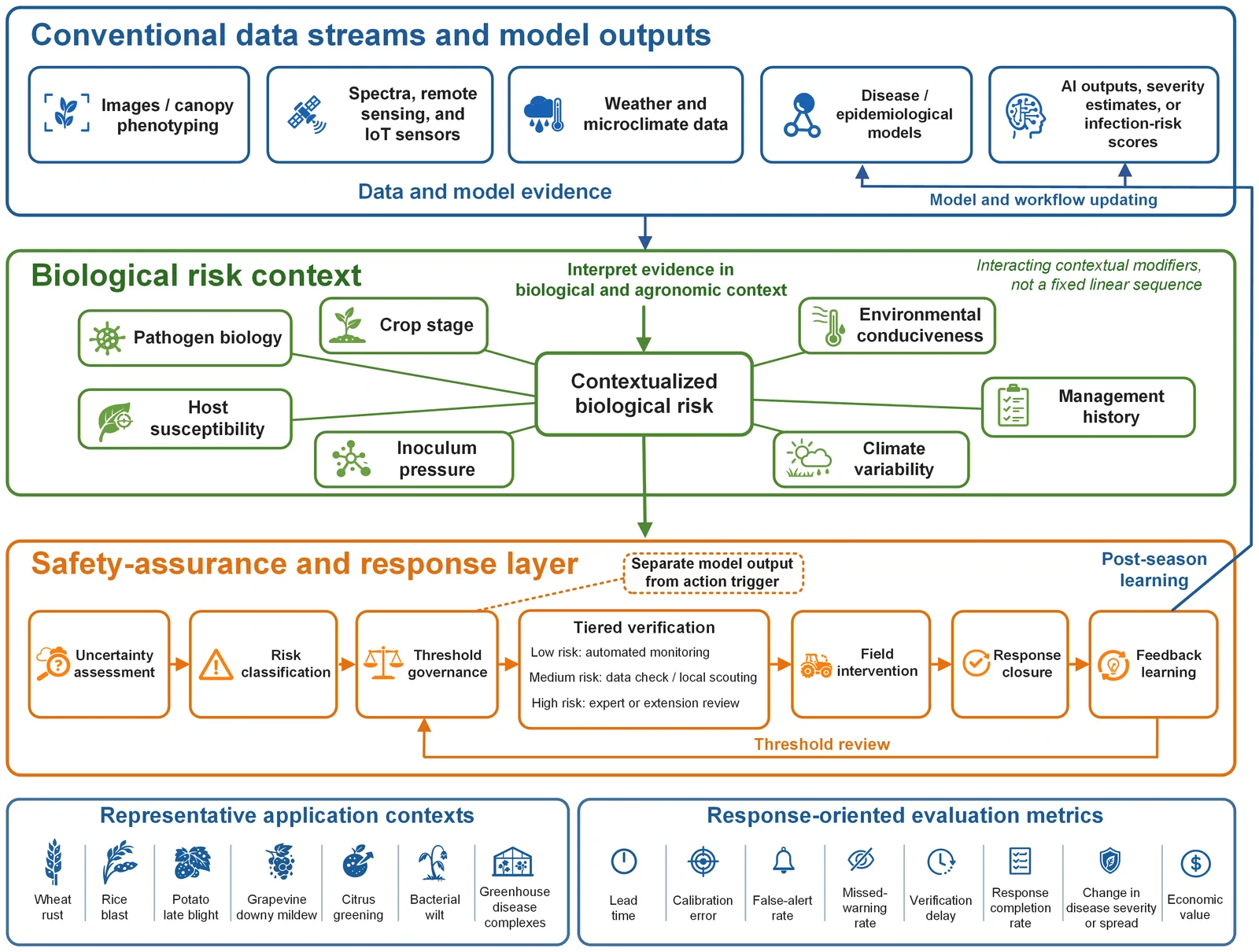
Safety-assured plant health early warning framework. The framework extends conventional AI detection and forecasting by adding biological risk context and a safety-assurance layer. Model outputs, sensor signals, weather-based forecasts, or infection-risk scores are interpreted together with interacting contextual modifiers: pathogen biology, host susceptibility, crop stage, environmental conduciveness, inoculum pressure, climate variability, and management history. These factors are not intended as a fixed linear sequence. The safety-assurance layer then supports uncertainty assessment, risk classification, threshold governance, tiered verification, field intervention, response closure, and feedback learning. Representative contexts include wheat rust, rice blast, potato late blight, grapevine downy mildew, citrus greening, bacterial wilt, and greenhouse disease complexes; response-oriented metrics include lead time, calibration, false alerts, missed warnings, verification delay, response completion, and disease or economic outcomes.

**Figure 1** summarizes the proposed framework. The upper layer recognizes conventional AI and forecasting outputs: images, spectra, sensors, weather data, disease models, probabilities, severity estimates, or infection-risk scores. The middle layer adds biological risk context: pathogen biology, host susceptibility, crop stage, environmental conduciveness, inoculum pressure, climate variability, and management history. These factors are interacting contextual modifiers rather than a fixed linear sequence. The lower layer converts contextualized outputs into risk classification, threshold decision, verification, intervention, response closure, and feedback learning. A key design principle is separation between model output and action trigger. The model may estimate disease probability or infection risk, whereas the warning threshold should reflect agronomic consequence, uncertainty, false-alarm and missed-infection costs, and the remaining opportunity for control.

Implementation must be proportional to production context. Large regional platforms may use automated weather feeds, remote sensing, calibrated uncertainty estimates, digital audit trails, and extension-service escalation. Smallholder or low-resource systems may begin with simpler rules: minimum image-quality checks, local scouting confirmation for high-consequence alerts, seasonal threshold review, and basic logging of false alerts and missed outbreaks. In large-scale agriculture, human-in-the-loop decision making should not mean that every signal requires expert review. A tiered approach is more realistic: low-risk signals can remain automated or monitored, medium-risk signals can trigger additional data collection, and high-consequence or uncertain signals can be escalated to experts or extension services.

Practical design also needs to consider computation at the edge, data ownership, communication coverage, compatibility with existing advisory services, and whether the expected reduction in losses or chemical use justifies the added monitoring workload. These conditions matter because a theoretically sound safety framework may fail if farmers do not trust alerts, if extension services cannot respond within the disease window, or if connectivity prevents timely transmission of field images and weather data. For this reason, validation should include not only laboratory or retrospective model performance, but also whether the warning could be understood, verified, and acted upon in the intended production system.

Evaluation should therefore include response-oriented indicators in addition to detection metrics. Useful indicators include lead time before visible outbreak or treatment deadline, calibration error, false-alert rate, missed-warning rate, verification delay, response completion rate, change in disease severity or spread, fungicide-use efficiency, economic benefit, user adoption and trust, robustness under degraded data conditions, and feedback-record quality. These indicators provide measurable criteria for testing whether safety-assurance design improves warning usefulness under real agricultural conditions. They also support comparison with established DSSs: a safety-assured workflow should be judged by whether it reduces harmful missed warnings without imposing unacceptable false-alert burden, labor demand, chemical use, or decision complexity.

**Table 1** translates the framework into implementation terms. Biological risk assessment is linked to calibration by risk class and lead time; FMEA to recurrent failure logs; threshold governance to false-alert and missed-warning rates; resilience to fallback activation and escalation time; and response closure to confirmation, intervention, follow-up, and post-season updates. Reporting these indicators would show whether warnings were delivered, correctly interpreted, and followed by appropriate management action. Studies could state in advance which action window, confirmation route, and management outcome define a useful warning. Negative findings would then reveal whether failure occurred in sensing, biological interpretation, threshold choice, communication, or field response.

**Table 1:** Safety-assurance elements, plant-pathology adaptation, implementation indicators, and representative application contexts for intelligent plant health early warning systems.

Safety-assurance element	Plant-pathology adaptation	Implementation and validation considerations	Representative application context
Biological risk assessment	Interprets AI or forecast output together with pathogen aggressiveness, host susceptibility, crop stage, weather conduciveness, inoculum pressure, management history, and action window.	Calibration by risk class; lead time before treatment deadline; documented false-negative tolerance.	Wheat rust verification under susceptible crop stage and humid weather; late blight risk under favorable weather (Chen, 2005; Small et al., 2015).
FMEA	Maps failure modes across data acquisition, disease biology, sensor function, model transfer, expert review, extension support, and field response.	Failure log completeness; recurrent failure modes; reduction in repeated data-quality or response failures.	Poor imagery, symptom ambiguity, missing data, delayed verification, or unimplemented treatment (Jafar et al., 2024; Zhao et al., 2025).
Bow-tie reasoning	Links host-pathogen-environment threats to missed or late warnings, preventive barriers, mitigative barriers, and outbreak consequences.	Presence and effectiveness of preventive and mitigative barriers; post-event review of barrier failures.	Wheat rust: inoculum pressure and conducive humidity leading to a missed warning and subsequent spread; barriers include scouting, verification, targeted treatment, and follow-up (Chen, 2005; Wellings, 2011).
Threshold governance	Defines when output becomes monitoring, verification, or action based on disease severity, crop stage, economic threshold, weather risk, uncertainty, and user capacity.	Threshold review frequency; false-alert rate; missed-warning rate; cost or fungicide-use efficiency.	Established disease DSS thresholds and adaptive verification under high-risk conditions (Helps et al., 2024; Yan et al., 2026).
Reliability and field validation	Tests whether the warning chain works across cultivars, regions, devices, seasons, disease stages, and management settings.	External validation performance; transfer limits; verification delay; robustness under sensor or weather-data gaps.	Rice blast across model types and intelligent disease detection across field conditions (Nettleton et al., 2019; Upadhyay et al., 2025).
Resilience and fallback monitoring	Maintains warning function when data quality, connectivity, weather feeds, or expert availability are degraded.	Fallback activation rate; time to escalation; continuity of monitoring during degraded conditions.	Backup scouting after uncertain detection and fallback monitoring under degraded data conditions (Jafar et al., 2024; Shoaib et al., 2025).
Response closure and learning	Records confirmation, intervention, follow-up inspection, disease outcome, and whether the warning was true, false, late, or missed.	Response completion rate; feedback record quality; post-season threshold or model updates.	DSS-guided management feedback and post-season learning (Helps et al., 2024).

## 6 Research gaps and future directions

First, field validation should report not only external performance but also where and why the warning chain fails. Such reports should include image-quality problems, ambiguous symptoms, model transfer limits, incomplete ground truth, poor connectivity, missing user response, and threshold decisions that were too sensitive or insufficiently protective. Negative or mixed results should be reported as evidence about the warning chain, not merely as evidence that a model was weak.

Second, uncertainty quantification should become more biologically interpretable. Future systems should distinguish uncertainty caused by sensor limitations or model shift from uncertainty caused by latent infection, pathogen race change, mixed stress physiology, evolving host resistance, or climate-driven changes in disease dynamics. Climate variability should also be incorporated into adaptive thresholds rather than treated only as background motivation.

Third, explainable AI should be connected to agronomic interpretation. Visual explanations are useful only if they show disease-relevant lesions, canopy zones, spectral patterns, or environmental drivers in ways that support expert verification and farmer trust (Barredo Arrieta et al., 2020; Selvaraju et al., 2020). Explanation should also support correction after false alerts: if a model repeatedly attends to soil background, water stains, or non-disease leaf damage, this should trigger data review and model revision.

Fourth, next-generation intelligent phytoprotection may combine foundation AI models, digital twins, federated learning, edge AI, autonomous scouting robots, synthetic training data, and multi-omics integration. These approaches could improve data coverage and biological specificity, but they also increase the need for version control, field validation, traceable threshold updates, and safeguards against amplifying local label noise or biased feedback.

Fifth, the value of safety-assured warning systems must be tested in crop-pathogen systems and production settings. Studies should compare conventional detection or forecasting workflows with workflows that add explicit uncertainty communication, risk-sensitive thresholds, tiered verification, response closure, and post-season learning. Such comparisons should include both technical outcomes and implementation outcomes, because a theoretically sound warning may still fail if data infrastructure, computation, extension support, validation protocols, or farmer incentives are insufficient.

## 7 Discussion and conclusion

AI, sensing, and remote monitoring have substantially improved the technical basis for plant disease and stress detection. The next challenge is to connect these outputs with plant-pathological risk, operational thresholds, verification pathways, and field response. Safety assurance is useful because it shifts attention from isolated model correctness to the dependability of the warning chain.

This perspective has practical implications: external validation before deployment, biological uncertainty reporting, documented threshold rules, tiered human-AI oversight, audit trails for field response, and learning from false alerts and missed outbreaks. It also has limitations. It is not a substitute for plant pathology, epidemiological modeling, integrated disease management, agronomic expertise, or rigorous machine-learning validation. Its value will depend on pathogen biology, crop type, production mode, data infrastructure, computational resources, extension capacity, farmer adoption, and economic feasibility.

Whether these added assurance functions improve existing warning systems remains to be established through comparative evaluation. These claims require comparative field studies, retrospective DSS analyses, and prospective deployment records. Useful comparisons should examine whether the added assurance layer improves decision timeliness, reduces damaging missed warnings without excessive false alerts, and strengthens post-season learning. Detection accuracy is one starting point; future systems should also support timely, proportionate, biologically interpretable, economically meaningful, and continuously learning action under field uncertainty.

## References

[B1] Araus J. L. Cairns J. E. (2014). Field high-throughput phenotyping: the new crop breeding frontier. Trends Plant Sci. 19, 52–61. doi: 10.1016/j.tplants.2013.09.008 24139902

[B2] Barbedo J. G. A. (2016). A review on the main challenges in automatic plant disease identification based on visible range images. Biosyst. Eng. 144, 52–60. doi: 10.1016/j.biosystemseng.2016.01.017 42574925

[B3] Barredo Arrieta A. Diaz-Rodriguez N. Del Ser J. Bennetot A. Tabik S. Barbado A. . (2020). Explainable artificial intelligence (XAI): concepts, taxonomies, opportunities and challenges toward responsible AI. Inf. Fusion. 58, 82–115. doi: 10.1016/j.inffus.2019.12.012 42574925

[B4] Chen X. M. (2005). Epidemiology and control of stripe rust [Puccinia striiformis f. sp. tritici] on wheat. Can. J. Plant Pathol. 27, 314–337. doi: 10.1080/07060660509507230 37339054

[B5] de Ruijter A. Guldenmund F. (2016). The bowtie method: A review. Saf. Sci. 88, 211–218. doi: 10.1016/j.ssci.2016.03.001 42574925

[B6] Dickstein E. R. Bocsanczy A. M. Champoiseau P. G. Jones J. B. Norman D. J. Paret M. . (2024). Recovery plan for Ralstonia solanacearum Race 3 Biovar 2 (Phylotype IIB, sequevars 1 and 2) causing brown rot of potato, bacterial wilt of tomato, and southern wilt of geranium. Plant Health Prog. 25, 98–139. doi: 10.1094/PHP-03-23-0027-RP

[B7] Furbank R. T. Tester M. (2011). Phenomics - technologies to relieve the phenotyping bottleneck. Trends Plant Sci. 16, 635–644. doi: 10.1016/j.tplants.2011.09.005 22074787

[B8] Garrett K. A. Forbes G. A. Savary S. Skelsey P. Sparks A. H. Valdivia C. (2011). Complexity in climate-change impacts: an analytical framework for effects mediated by plant disease. Plant Pathol. 60, 15–30. doi: 10.1111/j.1365-3059.2010.02409.x 40046247

[B9] Gawlikowski J. Tassi C. R. N. Ali M. Lee J. Humt M. Feng J. (2023). A survey of uncertainty in deep neural networks. Artif. Intell. Rev. 56, 1513–1589. doi: 10.1007/s10462-023-10562-9 30311153

[B10] Gottwald T. R. (2010). Current epidemiological understanding of citrus Huanglongbing. Annu. Rev. Phytopathol. 48, 119–139. doi: 10.1146/annurev-phyto-073009-114418 20415578

[B11] Guo C. Pleiss G. Sun Y. Weinberger K. Q. (2017). “ On calibration of modern neural networks”, in: Proceedings of the 34th International Conference on Machine Learning. Sydney, NSW: PMLR. 1321–1330.

[B12] Helps J. C. van den Bosch F. Paveley N. Jorgensen L. N. Holst N. Milne A. E. (2024). A framework for evaluating the value of agricultural pest management decision support systems. Eur. J. Plant Pathol. 169, 887–902. doi: 10.1007/s10658-024-02878-1 30311153

[B13] Hollnagel E. (2018). Safety-I and Safety-II: The Past and Future of Safety Management (Boca Raton, FL: CRC press).

[B14] Hollnagel E. Woods D. D. Leveson N. (2006). Resilience Engineering: Concepts and Precepts ( Aldershot, UK: Ashgate Publishing, Ltd).

[B15] IEC (2018). IEC 60812:2018 Failure modes and effects analysis (FMEA and FMECA) (Geneva: International Electrotechnical Commission).

[B16] IEC (2019). IEC 31010:2019 Risk management-Risk assessment techniques (Geneva: International Electrotechnical Commission).

[B17] ISO (2018). ISO 31000:2018 risk management - guidelines (Geneva: International Organization for Standardization).

[B18] Jafar A. Bibi N. Naqvi R. A. Sadeghi-Niaraki A. Jeong D. (2024). Revolutionizing agriculture with artificial intelligence: plant disease detection methods, applications, and their limitations. Front. Plant Sci. 15, 1356260. doi: 10.3389/fpls.2024.1356260 38545388 PMC10965613

[B19] Mahlein A. K. (2016). Plant disease detection by imaging sensors - parallels and specific demands for precision agriculture and plant phenotyping. Plant Dis. 100, 241–251. doi: 10.1094/PDIS-03-15-0340-FE 30694129

[B20] Mohanty S. P. Hughes D. P. Salathe M. (2016). Using deep learning for image-based plant disease detection. Front. Plant Sci. 7, 1419. doi: 10.3389/fpls.2016.01419 27713752 PMC5032846

[B21] Nettleton D. F. Katsantonis D. Kalaitzidis A. Sarafijanovic-Djukic N. Puigdollers P. Confalonieri R. (2019). Predicting rice blast disease: machine learning versus process-based models. BMC Bioinf. 20, 514. doi: 10.1186/s12859-019-3065-1 31640541 PMC6806664

[B22] Ovadia Y. Fertig E. Ren J. Nado Z. Sculley D. Nowozin S. (2019). “ Can you trust your model's uncertainty? Evaluating predictive uncertainty under dataset shift”, in: Advances in Neural Information Processing Systems 32. Red Hook, NY: Curran Associates, Inc. 13991–14002.

[B23] Ramcharan A. Baranowski K. McCloskey P. Ahmed B. Legg J. Hughes D. P. (2017). Deep learning for image-based cassava disease detection. Front. Plant Sci. 8, 1852. doi: 10.3389/fpls.2017.01852 29163582 PMC5663696

[B24] Rossi V. Salinari F. Poni S. Caffi T. Bettati T. (2014). Addressing the implementation problem in agricultural decision support systems: the example of vite.net. Comput. Electron. Agric. 100, 88–99. doi: 10.1016/j.compag.2013.10.011 42574925

[B25] Savary S. Willocquet L. Pethybridge S. J. Esker P. McRoberts N. Nelson A. (2019). The global burden of pathogens and pests on major food crops. Nat. Ecol. Evol. 3, 430–439. doi: 10.1038/s41559-018-0793-y 30718852

[B26] Selvaraju R. R. Cogswell M. Das A. Vedantam R. Parikh D. Batra D. (2020). Grad-CAM: visual explanations from deep networks via gradient-based localization. Int. J. Comput. Vision 128, 336–359. doi: 10.1007/s11263-019-01228-7 30311153

[B28] Shoaib M. S. M. Sadeghi-Niaraki A. Ali F. A. Hussain I. H. Khalid S. K. (2025). Leveraging deep learning for plant disease and pest detection: a comprehensive review and future directions. Front. Plant Sci. 16, 1538163. doi: 10.3389/fpls.2025.1538163 40061031 PMC11885274

[B27] Shtienberg D. (2013). Will decision-support systems be widely used for the management of plant diseases? Annu. Rev. Phytopathol. 51, 1–16. doi: 10.1146/annurev-phyto-082712-102244 23767845

[B29] Sishodia R. P. Ray R. L. Singh S. K. (2020). Applications of remote sensing in precision agriculture: A review. Remote Sens. 12, 3136. doi: 10.3390/rs12193136 30654563

[B30] Small I. M. Joseph L. Fry W. E. (2015). Development and implementation of the BlightPro decision support system for potato and tomato late blight management. Comput. Electron. Agric. 115, 57–65. doi: 10.1016/j.compag.2015.05.010 42574925

[B31] Strange R. N. Scott P. R. (2005). Plant disease: a threat to global food security. Annu. Rev. Phytopathol. 43, 83–116. doi: 10.1146/annurev.phyto.43.113004.133839 16078878

[B32] Upadhyay A. Chandel N. S. Singh K. P. Chakraborty S. K. Nandede B. M. Kumar M. . (2025). Deep learning and computer vision in plant disease detection: a comprehensive review of techniques, models, and trends in precision agriculture. Artif. Intell. Rev. 58, 92. doi: 10.1007/s10462-024-11100-x 30311153

[B33] Wellings C. R. (2011). Global status of stripe rust: a review of historical and current threats. Euphytica 179, 129–141. doi: 10.1007/s10681-011-0360-y 30311153

[B34] Yan J. Wu H. Diao Z. Miao Y. Zhang B. Zhao C. (2026). Recent developments and applications of crop disease detection, prediction, and early warning: A review. Engineering. 62 (7), 316–340. doi: 10.1016/j.eng.2025.10.032 42574925

[B35] Yuen J. (2002). Bayesian analysis of plant disease prediction. Plant Pathol. 51, 407–412. doi: 10.1046/j.0032-0862.2002.00741.x 37945311

[B36] Zhao J. Xu L. Ma Z. Li J. Wang X. Liu Y. . (2025). A review of plant leaf disease identification by deep learning algorithms. Front. Plant Sci. 16, 1637241. doi: 10.3389/fpls.2025.1637241 40909895 PMC12405175

